# Case Report: Potential benefits of a single functional neurology intervention in athletic rehabilitation and recovery: a case study

**DOI:** 10.3389/fspor.2024.1472948

**Published:** 2025-01-17

**Authors:** Jorge Rey-Mota, Guillermo Escribano-Colmena, Eduardo Navarro Jiménez, Carmen Cecilia Laborde-Cárdenas, Rodrigo Yáñez-Sepúlveda, Vicente Javier Clemente-Suárez

**Affiliations:** ^1^ Independent Researcher, Madrid, Spain; ^2^ Faculty of Health Sciences, Simón Bolívar University, Barranquilla, Colombia; ^3^ Vicerrectoría de Investigación e Innovación, Universidad Simón Bolívar, Barranquilla, Colombia; ^4^ Faculty of Education and Social Sciences, Universidad Andres Bello, Viña del Mar, Chile; ^5^ Faculty of Sports Sciences, Universidad Europea de Madrid, Madrid, Spain; ^6^ Grupo de Investigación en Cultura, Educación y Sociedad, Universidad de la Costa, Barranquilla, Colombia

**Keywords:** functional neurology, neuroplasticity, pain, sport performance, rehabilitation, thermography, fatigue

## Abstract

This study analyze the effect of a single functional neurology session on sports performance, pain perception, fatigue perception and thermographic muscular response in an international female weightlifting athlete with incapacity to tolerate weight. Functional neurology is a discipline leveraging neuroplasticity for non-invasive recovery and performance optimization. We found how a single session of functional neurology improved load tolerance, enabling her to lift weights at pre-injury levels after just a single session of functional neurology and reduced pain perception from 80 to 0 and fatigue perception from 90 to 20 on a 100-point scale while thermographic data showed up to a 15% decrease in body temperature indicating reduced inflammation and improved muscle recovery. Then, we found how a single functional neurology intervention produced an improve in sports performance, pain and fatigue perceptions, and peripheral vascular response in an inter-national female weightlifting athlete with an initial incapacity to tolerate weight. This supports the incorporation of functional neurology into sports rehabilitation, under-scoring its potential in optimizing athletes' health and performance.

## 1 Introduction

Functional neurology represents a pivotal shift in the approach to healthcare for international athletes, leveraging the inherent plasticity of the nervous system to optimize performance and recovery. This discipline, emerging from the broader field of chiropractic medicine, emphasizes a paradigm where the functionality of the nervous system is central to overall health and athletic performance. Unlike traditional medical treatments, functional neurology avoids pharmaceuticals and invasive procedures in favor of manipulative therapies and sensory-based interventions designed to stimulate neuroplastic changes within the brain and nervous system (1, 2). These interventions capitalize on the capacity of the nervous system to adapt and reorganize throughout life, challenging the outdated notion that significant neuroplasticity is confined to early developmental stages (2).

In the domain of high-performance sports, the demands on an athlete's nervous system are immense and multifaceted, making functional neurology particularly relevant (3–5). Athletes constantly seek innovative strategies to enhance their performance, minimize injury risk, and accelerate recovery. Functional neurology addresses these needs by targeting neurological dysfunctions through specific interventions designed to improve balance, proprioception, and vestibular function, which are fundamental for maintaining equilibrium, coordinating movements, and executing complex maneuvers with precision (6, 7).

The use of infrared thermography (IRT) in sports medicine complements this approach, offering a non-invasive technique to monitor physiological responses, detect thermal imbalances, and identify areas of potential injury (8, 9). IRT has demonstrated utility in analyzing temperature distribution patterns, which correlate with inflammation or muscular stress, enabling practitioners to identify areas at risk and customize training regimens accordingly (8, 10). Recent studies have highlighted its potential to detect early signs of stress, inflammation, and muscle imbalance, underscoring its growing role in sports rehabilitation and injury prevention (11, 12). Moreover, IRT provides a valuable tool for evaluating the effectiveness of interventions, such as functional neurology, by monitoring changes in skin temperature that may reflect underlying physiological and neurological adaptations (13).

Despite its potential, functional neurology remains underexplored in the athletic population. While studies have demonstrated the efficacy of neuroplasticity-based therapies in addressing chronic pain and enhancing musculoskeletal function (14, 15), there is limited evidence regarding their application in elite sports settings. The integration of functional neurology with non-invasive diagnostic tools like IRT offers an opportunity to advance the understanding of these interventions in sports medicine. This study aims to analyze the effects of a single session of functional neurology on sports performance, pain perception, fatigue perception, and peripheral vascular response in an elite female weightlifting athlete unable to tolerate load. The initial hypothesis is that a single session of functional neurology may enhance sports performance, reduce pain and fatigue perceptions, and improve peripheral vascular response. The findings of this case study seek to provide preliminary insights into the potential benefits of functional neurology, bridging the gap between theoretical frameworks and practical applications in high-performance sports (16, 17).

## 2 Methods

### 2.1 Participant

The study focused on a 30-year-old female elite weightlifting athlete with a height of 160 cm and a weight of 60 kg. The participant had been competing at the international level for over 20 years and was an active member of the national weightlifting team since 2008. Her athletic career included significant achievements, such as being a 14-time National Champion, holding more than 10 national records, and securing podium finishes in events like the Junior European Championships, Senior European Championships, and Mediterranean Games. The athlete presented to the clinic with a primary complaint of severe, persistent pain in the left hip, which had progressively worsened over the past three months, leading to an inability to perform lifts involving significant weight. Her specific performance metrics prior to the onset of symptoms included a personal best of 83 kg in the snatch and 94 kg in the clean and jerk. The patient reported unsuccessful outcomes from previous interventions, including a 6-week physiotherapy program involving manual therapy, targeted exercises, and electrostimulation therapy. These interventions provided only temporary symptom relief and failed to restore her ability to train or compete effectively. She also disclosed a psychosocial impact, expressing frustration and anxiety about her declining performance and the potential impact on her career. A detailed medical history revealed no prior surgeries or significant injuries apart from minor shoulder strains managed conservatively in the past. The patient denied any family history of musculoskeletal or neurological disorders. A baseline psychological assessment indicated mild levels of distress, attributed to her prolonged recovery and competitive pressures. The participant provided written informed consent to participate in the study and to publish the anonymized data, which adhered to the ethical standards outlined in the Declaration of Helsinki and received ethical approval from the University's Bioethics Committee under code 2024-738.

### 2.2 Procedure

Before and after the functional neurology intervention, we analyzed the following variables:.

**Figure 1:**
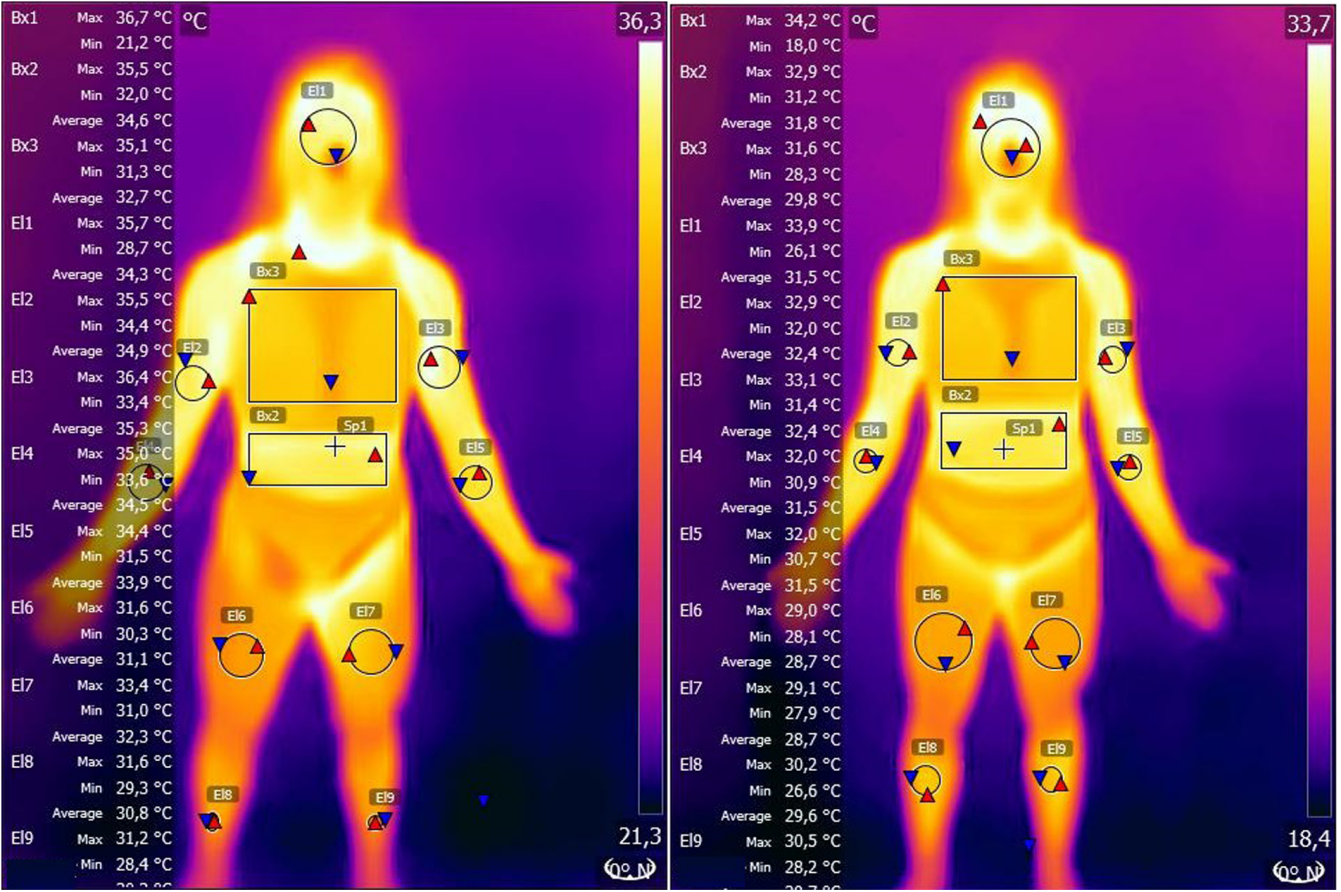
Thermographic modifications before and after the functional neurology treatment in the different body regions.

**Figure 2:**
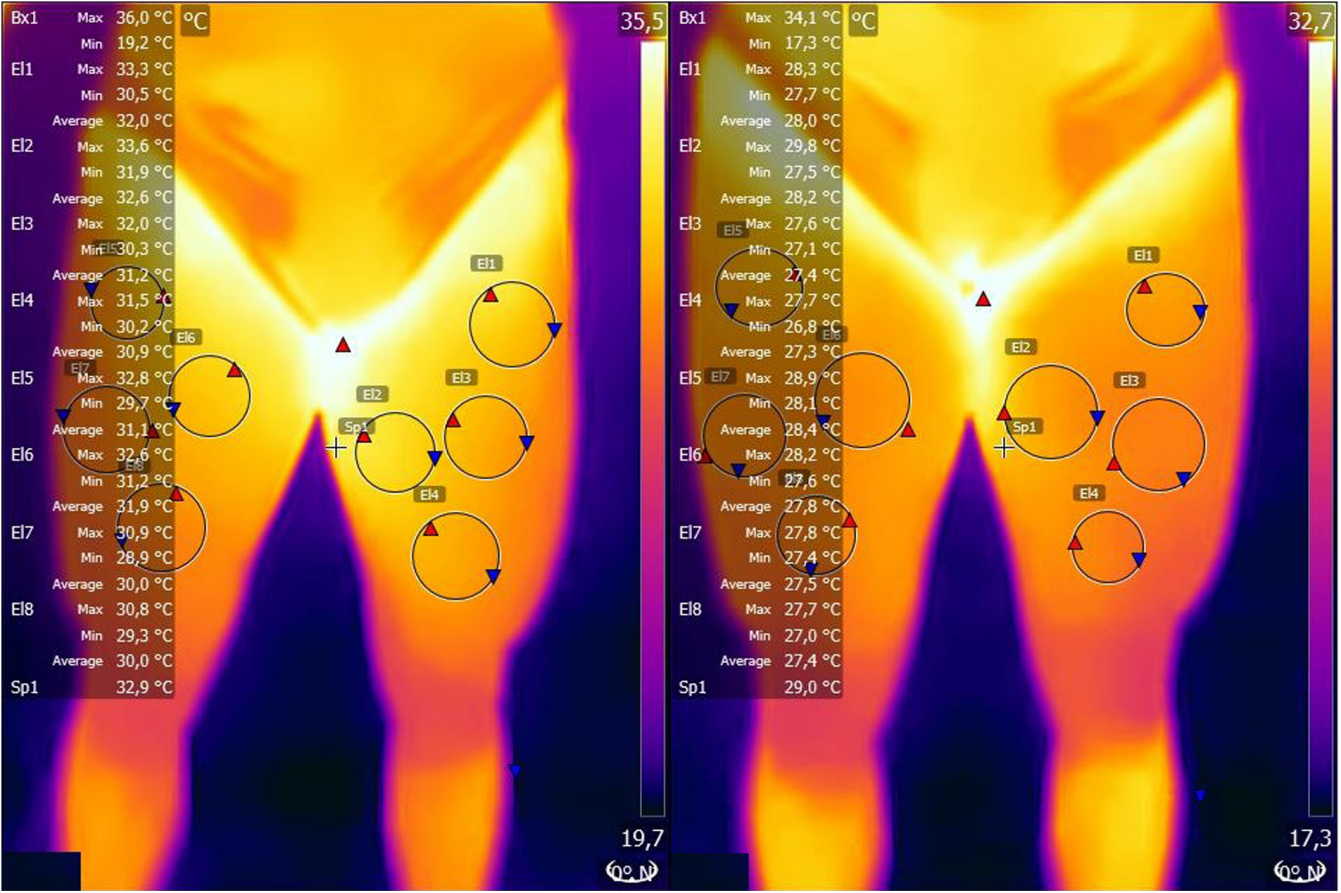
Thermographic modifications before and after the functional neurology treatment in the athlete hip.

1. Peripheral Vascular Response: Assessed through infrared thermography, capturing changes in blood flow and inflammation indicators. Thermographic images were captured in line with the European Association of Thermology's guidelines (13). The thermographic images were captured of the anterior body and thigh regions. The models for thermographic analysis are presented in Figures 1, 2. Maximal, minimal, and average temperatures were quantified in each specified region delineated in the Figures 1, 2. The thermographic data acquisition was performed using a FLIR ONE Edge Pro (Teledyne FLIR, Oregon, USA), while the analysis of the thermal imagery was conducted utilizing FLIR Tools software. By analyzing these thermographic images, we were able to monitor the distribution of blood flow in various body regions before and after the functional neurology intervention. This method allowed us to quantify the maximal, minimal, and average temperatures in specific areas, which are indicative of the underlying vascular activity. Higher temperatures generally correspond to increased blood flow and potential inflammation, whereas lower temperatures suggest reduced blood flow and inflammation. Through these measurements, we provided objective data on the functionality of blood vessels and flow rates, highlighting the physiological impacts of the intervention on the athlete's vascular system (16).
2. Pain Perception: Measured with a 0-100 visual analoge scale (VSA) to quantify changes in the athlete's experienced pain levels (18)
3. Fatigue Perception: Assessed with a 0-100 VSA to evaluate alterations in the athlete's reported fatigue levels (19)

Measurements were conducted in an environmentally controlled room, maintaining a stable temperature of 22.3°C and a humidity level of 41.4%.

### 2.3 Functional neurology intervention

The intervention was designed following a comprehensive clinical assessment of the participant's neurological and psychological status. This included evaluations of verbal processing, visual tests for eye tracking and oculomotor reflexes, proprioceptive and motor coordination assessments. This thorough evaluation guided the planning of a tailored neuromodulatory intervention (17, 20). The functional neurology session incorporated evidence-based techniques aimed at enhancing autonomic balance and neuroplasticity. These techniques were adapted from the ®NeuroReEvolution methodology and targeted specific neurological dysfunctions identified during the assessment. The intervention components were as follows:

1. Vestibular Stimulation: Controlled head movements and positional changes were performed to integrate vestibular inputs with autonomic and brainstem circuits. These movements aimed to improve balance, coordination, and autonomic regulation (21).
2. Ocular Reflex Activation: Eye-tracking exercises were used to stimulate oculomotor reflexes and enhance visual-motor integration. These guided tasks targeted the improvement of neurological pathways linked to eye movement control and cognitive processing (22).
3. Proprioceptive Inputs: Gentle tactile stimulation was applied to mechanoreceptive areas, such as the hands and feet, to enhance sensory feedback and neural integration. These inputs aimed to recalibrate proprioceptive signals and improve motor coordination (23).
4. Neurovisceral Reflex Activation: Light pressure was applied to neurovascular points to stimulate parasympathetic pathways, promoting vagal tone and reducing sympathetic dominance. This technique sought to modulate autonomic balance and enhance the participant's recovery and relaxation responses (22).

The intervention was implemented with a focus on leveraging neuroplasticity to optimize autonomic function, reduce psychological distress, and restore motor coordination. These techniques demonstrated potential for modulating HRV, reducing sympathetic dominance, and promoting parasympathetic activity, addressing the participant's specific needs. By incorporating these approaches, the session provided an integrated neuromodulatory strategy to address the participant's dysregulated autonomic and motor systems while offering immediate benefits in terms of recovery and rehabilitation.

### 2.4 Diagnostic assessment

The participant, an elite weightlifter, presented to the clinic with complaints of significant pain in her hip, alongside an inability to perform lifts involving substantial weights. This condition, marked by a noticeable intolerance to load and associated discomfort, had increasingly impeded her training and competitive performance. Her specific performance metrics, including a snatch record of 83 kg and a clean and jerk of 94 kg within her weight class, underscore her high standing in the weightlifting community, despite these challenges. A comprehensive diagnostic assessment was performed to identify underlying neurological, autonomic, and musculoskeletal imbalances. The assessment consisted of:

1. Neurological Evaluation:
  ○ Verbal and cognitive processing tests were conducted to identify potential delays or asymmetries in hemispheric activity.
  ○ Eye-tracking and oculomotor reflex tests were performed to evaluate the integration of visual-motor pathways, revealing mild delays in saccadic movements and a dysregulated vestibulo-ocular reflex.
2. Autonomic Function Testing:
  ○ Heart Rate Variability (HRV) analysis was employed as a non-invasive measure of autonomic balance, revealing reduced parasympathetic tone and heightened sympathetic dominance, consistent with prolonged physical and psychological stress.
3. Proprioceptive and Motor Coordination Assessments:
  ○ Joint position sense tests and balance evaluations highlighted proprioceptive deficits, particularly in the left hip and lower limb, which were associated with compensatory motor patterns.
4. Thermographic Imaging:
5. Infrared thermography (IRT) was used to assess skin temperature asymmetries, revealing elevated temperatures in the left hip and thigh region indicative of localized inflammation.

#### 2.4.1 Challenges and reasoning

• Challenges:
  ○ The primary diagnostic challenge was differentiating between structural musculoskeletal dysfunctions and neurological dysregulations contributing to the participant's symptoms.
  ○ The lack of prior functional neurology applications in similar cases limited the availability of comparative data for the assessment and intervention.
• Reasoning:
  ○ The diagnostic strategy focused on identifying neurological imbalances and autonomic dysfunctions that could exacerbate musculoskeletal pain and fatigue. By addressing these root causes through neuromodulation, the intervention sought to provide long-term functional recovery.

## 3 Results and discussion

This case study highlights the potential benefits of functional neurology interventions in addressing the complex demands of elite sports rehabilitation and recovery. The participant demonstrated a significant improvement in load tolerance, enabling her to return to pre-injury performance levels following a single 45 min session. This rapid recovery contrasts with traditional rehabilitation approaches, which often require multiple sessions over weeks or months to achieve similar outcomes (24).

The thermographic analysis revealed a significant reduction in skin temperature across various body regions post-intervention, suggesting a decrease in inflammation and improved vascular function (Tables 1, 2). Elevated skin temperatures are typically indicative of localized inflammation or stress, while reductions may reflect recovery and homeostasis (25). This aligns with prior research demonstrating the efficacy of infrared thermography (IRT) in monitoring physiological responses and identifying stress patterns in athletes (11, 26). For instance, studies have established that thermographic asymmetries can precede injury onset, making IRT a valuable tool for both preventive and evaluative purposes (27, 28). The observed changes in temperature distribution in this study suggest that the functional neurology intervention facilitated vascular and metabolic adaptations conducive to recovery. In addition to thermographic changes, the participant reported a complete resolution of pain, with a reduction from 80 to 0 on a 100-point scale, and a significant decrease in fatigue perception, from 90 to 20. These outcomes are consistent with the principles of functional neurology, which emphasize the modulation of neuroplasticity to address pain and dysfunction (1, 29). Pain reduction can be attributed to the recalibration of maladaptive neural pathways through targeted interventions, such as proprioceptive and vestibular stimulation (2, 30). By disrupting aberrant neural feedback loops, functional neurology interventions enhance the nervous system's capacity to adapt and restore function (31).

**Table 1:** Modification of temperature (°C) before and after the functional neurology intervention in different body regions.

Anatomical Region	Pre			Post			% Change		
	Max	Min	Avg	Max	Min	Avg	Max	Min	Avg
Head	35.7	28.7	34.3	33.9	26.1	31.5	−5.0	−9.1	−8.2
Right Arm	35.5	34.4	34.9	32.9	32.0	32.4	−7.3	−7.0	−7.2
Left Arm	36.4	33.4	35.3	33.1	31.4	32.4	−9.1	−6.0	−8.2
Right Forearm	35.0	33.6	34.5	32.0	30.9	31.5	−8.6	−8.0	−8.7
Left Forearm	34.4	31.5	33.9	32.0	30.7	31.5	−7.0	−2.5	−7.1
Abdomen	35.5	32.0	34.6	32.9	31.2	31.8	−7.3	−2.5	−8.1
Chest	35.1	31.3	32.7	31.6	28.3	29.8	−10.0	−9.6	−8.9
Right Thigh	33.4	31.0	32.3	29.1	27.9	28.7	−12.9	−11.1	−7.7
Left Thigh	31.6	29.3	30.8	30.2	26.6	29.6	−4.4	−9.2	−3.9
Right Calf	31.2	28.4	30.3	30.5	28.2	29.7	−2.2	−0.7	−2.0
Left Calf	31.2	28.4	30.3	30.5	28.2	29.7	−2.2	−0.7	−2.0

**Table 2:** Modification of temperature (°C) before and after the functional neurology intervention in thighs.

Region	Pre Max	Pre Min	Pre Avg	Post Max	Post Min	Post Avg	% Change Max	% Change Min	% Change Avg
Right Upper Leg	33.3	30.5	32.0	28.3	27.7	28.0	−15.0	−9.2	−12.5
Right Inner Leg	33.6	31.9	32.6	29.8	27.5	28.2	−11.3	−13.8	−13.5
Right Outer Leg	32.0	30.3	31.2	27.6	27.1	27.4	−13.8	−10.6	−12.2
Right Lower Leg	31.5	30.2	30.9	27.7	26.8	27.3	−12.1	−11.3	−11.7
Left Upper Leg	30.9	32.8	31.1	28.9	28.1	28.4	−11.7	−5.4	−8.7
Left Inner Leg	31.1	32.6	31.9	28.4	27.6	27.8	−8.7	−13.5	−12.9
Left Outer Leg	30.9	28.9	30.0	27.8	27.4	27.5	−10.0	−5.2	−8.3
Left Lower Leg	30.8	29.3	30.0	27.7	27.0	27.4	−10.1	−7.8	−8.7

The significant decrease in fatigue perception observed in this study may reflect improvements in autonomic balance. Fatigue is often associated with dysregulation of the hypothalamic-pituitary-adrenal (HPA) axis and increased sympathetic activity, which can lead to heightened energy expenditure and impaired recovery (32, 33). By incorporating neuromodulatory techniques such as vestibular stimulation and neurovisceral reflex activation, the intervention likely promoted parasympathetic activity and reduced sympathetic dominance, as evidenced by prior studies linking such techniques to HRV modulation (17, 34).

The results also underscore the importance of incorporating non-invasive, neuroplasticity-based methods into elite sports rehabilitation. Traditional physiotherapy and recovery strategies, while effective, often rely on passive or repetitive protocols that may not adequately address the neurological underpinnings of pain and fatigue (24, 35). In contrast, functional neurology leverages a systemic approach to optimize the interplay between the nervous system and the musculoskeletal system. This approach is particularly relevant for high-performance athletes, where the integration of sensory, motor, and autonomic systems is critical for peak performance (5, 7).

The integration of IRT as a diagnostic and evaluative tool in this study complements the principles of functional neurology by providing objective evidence of physiological changes. Prior studies have demonstrated the utility of IRT in identifying thermal asymmetries associated with inflammation and muscle stress, highlighting its role in sports medicine for monitoring training loads and recovery (11, 26, 27). For example, Marins et al. (25) discussed how reduced thermographic temperatures following intervention might indicate a decrease in metabolic activity and inflammation, aligning with the findings in this case. Furthermore, Hildebrandt et al. (24) emphasized the relevance of IRT in detecting early signs of musculoskeletal stress, supporting the observed improvements in this study.

The participant's pain reduction can also be contextualized within the broader framework of neuroplasticity-driven interventions. Neuroplastic changes induced by functional neurology have been shown to recalibrate the nervous system's response to pain, reducing maladaptive feedback and restoring functional balance (29, 30). Stone et al. (31) and Pelletier et al. (34) demonstrated that targeted sensory and proprioceptive stimuli could disrupt entrenched nociceptive pathways, allowing for the formation of healthier neural circuits. This mechanism likely contributed to the participant's rapid improvement in pain perception and overall functionality.

Another critical finding was the significant reduction in fatigue perception, which can be linked to improvements in autonomic regulation. Fatigue in elite athletes often stems from an imbalance in autonomic function, particularly an overactive sympathetic response and inadequate parasympathetic recovery (32, 33). By employing techniques such as vestibular stimulation and neurovisceral reflex activation, the intervention facilitated autonomic balance, likely reducing the participant's energy expenditure associated with compensatory mechanisms (17, 34). Studies by Shaffer and Ginsberg (17) have underscored the impact of neuromodulatory techniques on HRV improvement, reflecting enhanced parasympathetic tone and reduced physiological stress.

The broader implications of these findings suggest that functional neurology could play a transformative role in sports rehabilitation by offering a systemic and individualized approach to recovery. Unlike conventional methods that often address symptoms in isolation, functional neurology integrates sensory, motor, and autonomic interventions to target the root causes of dysfunction. This approach aligns with emerging research advocating for multidisciplinary rehabilitation protocols that incorporate neuroplasticity-based therapies (5, 24). However, the results must be interpreted with caution, as this study focused on a single case without longitudinal follow-up or a control group, limiting the generalizability of the findings.

Future research should aim to validate these preliminary results through randomized controlled trials and longitudinal studies that assess the efficacy of functional neurology interventions across diverse athletic populations. Additionally, incorporating objective biomarkers, such as HRV, inflammatory markers, and neuroimaging data, could provide further insights into the mechanisms underlying the observed benefits. The use of IRT as a monitoring tool in such studies could also enhance the understanding of physiological changes associated with functional neurology. This case study illustrates the potential of functional neurology as a non-invasive and neuroplasticity-driven intervention for elite athletes, addressing key aspects of pain management, fatigue reduction, and physiological recovery. While the results are promising, further research is needed to establish standardized protocols and determine the long-term impact of these interventions on athletic performance and rehabilitation.

Further research into the mechanisms underlying these observed changes, including the role of neuroplasticity in mediating the effects of functional neurology interventions, will be crucial in advancing our understanding of how these approaches can be optimized to support pain management and rehabilitation. The exploration of thermographic changes as a proxy for physiological improvements offers a valuable tool for quantifying the impact of these interventions, providing a bridge between subjective experiences of pain relief and objective evidence of physiological change.

### 3.1 Follow-up and outcomes

#### 3.1.1 Clinician-assessed outcomes

The intervention was evaluated using objective metrics, including infrared thermography (IRT), pain perception, and fatigue perception scales. Post-intervention IRT data showed a 10%–15% reduction in skin temperature across the left hip and thigh regions, suggesting reduced inflammation and improved vascular response. The clinician also noted improvements in joint stability and proprioceptive function during follow-up assessments conducted one and three weeks after the intervention. These assessments confirmed sustained improvements in motor coordination and functional load tolerance.

#### 3.1.2 Patient-assessed outcomes

The participant reported a complete resolution of pain within 24 h post-intervention, with Visual Analogue Scale (VAS) scores decreasing from 80 to 0. Fatigue perception, measured using a 100-point scale, reduced from 90 to 20. Additionally, the patient expressed psychological relief and increased confidence in returning to competition, highlighting the intervention's positive impact on both physical and emotional well-being.

#### 3.1.3 Intervention adherence

The participant complied fully with the intervention protocol, including the prescribed rest and gradual reintroduction to weightlifting exercises following the session. Adherence was facilitated by detailed guidance provided by the clinician and regular check-ins to monitor progress.

#### 3.1.4 Adverse and unanticipated events

No adverse or unanticipated events were reported during or after the intervention. The participant experienced no discomfort or side effects, and follow-up sessions confirmed the absence of any negative outcomes.

#### 3.1.5 Patient perspective

The patient expressed profound relief and satisfaction with the outcomes of the intervention. Within 24 h of the session, she reported a complete resolution of hip pain and a significant reduction in fatigue, describing the experience as “transformative” for her athletic performance and overall well-being. She noted that the absence of pain allowed her to regain confidence in her ability to return to high-level competition, which had been severely impacted by her prolonged injury and prior failed rehabilitation attempts. The participant also highlighted the immediate improvement in her physical comfort and psychological state, stating that the intervention provided “a renewed sense of hope and control” over her recovery process. She found the non-invasive nature of the treatment particularly appealing and expressed enthusiasm for the integration of such approaches into sports medicine. Overall, the patient regarded the intervention as a pivotal moment in her rehabilitation journey, attributing her rapid recovery and emotional resilience to the targeted and individualized nature of the functional neurology techniques employed.

## 4 Conclusion

This case study demonstrates the potential of functional neurology as an innovative, non-invasive approach to sports rehabilitation, yielding significant improvements in pain perception, fatigue, and thermographic indicators of inflammation and vascular response after a single tailored session. By leveraging neuroplasticity and autonomic modulation, the intervention addressed underlying dysfunctions, offering promising preliminary results for recovery and rehabilitation in elite athletes. However, the findings should be interpreted cautiously due to the single-case design and lack of longitudinal follow-up. Future research with larger samples, standardized protocols, and objective biomarkers is needed to validate these results and explore the broader applications of functional neurology in sports medicine.

## Data Availability

The original contributions presented in the study are included in the article/Supplementary Material, further inquiries can be directed to the corresponding authors.
